# Clarification on Host Range of *Didymella pinodes* the Causal Agent of Pea Ascochyta Blight

**DOI:** 10.3389/fpls.2016.00592

**Published:** 2016-05-13

**Authors:** Eleonora Barilli, Maria José Cobos, Diego Rubiales

**Affiliations:** Institute for Sustainable AgricultureCSIC, Córdoba, Spain

**Keywords:** pea, legume, ascochyta blight, dydimella pinodes, host range, disease management

## Abstract

*Didymella pinodes* is the principal causal agent of ascochyta blight, one of the most important fungal diseases of pea (*Pisum sativum*) worldwide. Understanding its host specificity has crucial implications in epidemiology and management; however, this has not been clearly delineated yet. In this study we attempt to clarify the host range of *D. pinodes* and to compare it with that of other close *Didymella* spp. *D. pinodes* was very virulent on pea accessions, although differences in virulence were identified among isolates. On the contrary, studied isolates of *D. fabae, D. rabiei*, and *D. lentil* showed a reduced ability to infect pea not causing macroscopically visible symptoms on any of the pea accessions tested. *D. pinodes* isolates were also infective to some extend on almost all species tested including species such as *Hedysarum coronarium, Lathyrus sativus, Lupinus albus, Medicago* spp., *Trifolium* spp., *Trigonella foenum-graecum*, and *Vicia articulata* which were not mentioned before as hosts of *D. pinodes*. On the contrary, *D. lentil* and *D. rabiei* were more specific, infecting only lentil and chickpea, respectively. *D. fabae* was intermediate, infecting mainly faba bean, but also slightly other species such as *Glycine max, Phaseolus vulgaris, Trifolium* spp., *Vicia sativa*, and *V. articulata*. DNA sequence analysis of the nuclear ribosomal internal transcribed spacer region (ITS) was performed to confirm identity of the isolates studies and to determine phylogenetic relationship among the *Didymella* species, revealing the presence of two clearly distinct clades. Clade one was represented by two supported subclusters including *D. fabae* isolates as well as *D. rabiei* with *D. lentil* isolates. Clade two was the largest and included all the *D. pinodes* isolates as well as *Phoma medicaginis* var. *pinodella*. Genetic distance between *D. pinodes* and the other *Didymella* spp. isolates was not correlated with overall differences in pathogenicity. Based on evidences presented here, *D. pinodes* is not specialized on pea and its host range is larger than that of *D. fabae, D. lentil*, and *D. rabiei*. This has relevant implications in epidemiology and control as these species might act as alternative hosts for *D. pinodes*.

## Introduction

Cool season legumes play an important role in farming systems worldwide (Siddique et al., 2012). They provide important services to societies as they are important sources of oil, fiber, protein-rich food and feed while supplying nitrogen (N) to agro-ecosystems via their unique ability to fix atmospheric N_2_ in symbiosis with the soil bacteria rhizobia, increasing soil carbon content, and stimulating the productivity of the crops that follow (Jensen et al., 2012). Among them, field pea (*Pisum sativum* L.) is widely grown across cooler temperate zones of the world on about 6.2 m ha annually with total production generally ranging between 10 and 11 m tons (FAOSTAT, 2015).

Ascochyta blight diseases represent serious limitations to legume production worldwide (Rubiales and Fondevilla, 2012; Khan et al., 2013). *Didymella fabae* Jellis and Punith. (anamorph *Ascochyta fabae* Speg.), *D. lentis* Kaiser, Wang and Rogers (anamorph *A. lentis* Vassiljevsky) and *D. rabiei* (Kovachevski) v. Arx (anamorph *A. rabiei* (Pass) Labr.) are the causal agents of ascochyta blights on faba bean (*Vicia faba* L.), lentil (*Lens culinaris* Medik.), and chickpea (*Cicer arietinum* L.), respectively (Kaiser et al., 1997; Hernandez-Bello et al., 2006; Tivoli and Banniza, 2007). Yield losses caused by aschochyta blight are in order of 40% in lentil (Gossen and Derksen, 2003), but in severe cases losses higher than 90% have been reported in faba bean (Omri Benyoussef et al., 2012) and chickpea (Pande et al., 2005). In pea, this disease is caused by a complex of fungi formed by *Ascochyta pisi* Lib., *Didymella pinodes* (Berk and Blox) Petrak, *Phoma medicaginis* var. *pinodella* (L.K. Jones) Morgan-Jones and K.B. Burch and *Phoma koolunga* Davidson, Hartley, Priest, Krysinska-Kaczmarek, Herdina, McKay, and Scott (this last is, at the time, with limited presence in South and Western Australia; Tran et al., 2016). Of these, *D. pinodes* (formerly known as *Mycosphaerella pinodes* (Berk. and A. Bloxam) Vestergr., anamorph *Ascochyta pinodes* L.K. Jones) is the most predominant and damaging pathogen and under some conditions can cause yield losses up to 70% (Tivoli and Banniza, 2007).

*D. pinodes* remains an extremely difficult pathogen to control, primarily due to limited levels of host resistance available, and secondarily because fungicides are often uneconomic (Khan et al., 2013). Therefore, the main disease control strategy has been to avoid sowing close to infested field pea stubbles and/or to delay sowing of field pea crops for as long as possible in order to avoid the majority of ascospores, particularly those falling on emerging pea seedlings (Salam et al., 2011). Nevertheless, the late sowing is not an option in some countries due to the short crop season and this practice incurs unsustainable yield penalties in many instances (Khan et al., 2013). Other control measures involving crop rotation and intercropping have been also tested (Bailey et al., 2001; McDonald and Peck, 2009; Fernández-Aparicio et al., 2010) showing potential in disease reduction.

A better understanding of a pathogen's host range is critical to handle ascochyta blight and to break its cycle with more effectiveness, particularly in regions where pea is frequently grown and where the disease is endemic or where ascospores are an overriding primary source of initial infection. *D. pinodes* is known to be less specialized than other *Didymella* spp. (Sprague, 1929; Sattar, 1934; Le May et al., 2014), which increases the potential of this specie to survive. In fact, adjacent naturally infected alternative hosts could serve as important sources of inoculum to initiate disease epidemics on cultivated peas. So, the impact of alternative hosts on plant pathogen adaptation must be taken into account since they affect the survival of pathogen populations, and transmission opportunities to different components and ecological niches (wild/cultivated, cultivated/cultivated; Woolhouse et al., 2001), as recently showed for *D. rabiei* (Trapero-Casas and Kaiser, 2009). Nevertheless, despite its importance, the host range of *D. pinodes* on legume species other than *Pisum* spp. is poorly understood (Bretag, 2004; Taylor and Ford, 2007; Khan et al., 2013; Le May et al., 2014).

The aims of this study were therefore (i) to further refine the host range of *D. pinodes* within cultivated and wild legumes; (ii) to assess the susceptibility/resistance of different accessions within each of these legume species to nine isolates of *D. pinodes* from different geographical origin; (iii) to compare the host range of *D. pinodes* with that of other *Didymella* species; and (iv) to relate fungal isolates by ITS molecular markers.

## Materials and methods

### Fungal isolates

Nine isolates of *D. pinodes*, two isolates of *D. fabae*, one of *D. lentil*, and one of *D. rabiei*, all from IAS-CSIC fungal collection, were used in the experiments (information reported in Table 1). Local *D. pinodes* isolate Dp-CO-99, as well as isolates Dp-FR-88, Dp-PO-03 and Dp-JAP-03 have previously shown to differ in aggressiveness toward pea accessions (Fondevilla et al., 2005). All isolates were monoconidial and were preserved in sterile cellulose filter papers.

**Table 1:** **Codes of reference, specie definition, collecting site, year and GenBank accession relative to the fungus isolates used in the study**.

**Fungal code**	**Fungal specie**	**Collecting site**	**Collecting year**	**GenBank n°**
Dp-CO-99	*Didymella pinodes*	Córdoba, Spain	1999	KR259388
Dp-FR-88	*D. pinodes*	Rennes, France	2003	KR259380
Dp-PdT-03	*D. pinodes*	Palmar de Troya, Spain	2003	KR259391
Dp-PO-03	*D. pinodes*	Wa̧sy, Poland	2003	KR259387
Dp-JAP-03	*D. pinodes*	Japan	2003	KR259392
Dp-ANN-13	*D. pinodes*	Annaba, Algeria	2013	KR259390
Dp-M07-4	*D. pinodes*	Perth, Australia	2013	KR259383
Dp-Esc-13	*D. pinodes*	Escacena del Campo, Spain	2013	KR259389
Dp-KHM-13	*D. pinodes*	Khemis Miliana, Algeria	2013	KR259386
Df-AU04	*D. fabae*	Gleisdorf, Austria	2005	KR259385
Df-857	*D. fabae*	France	2005	KR259384
Dl-AL10	*D. lentil*	Germany	2010	KR259381
Dr-Pt04	*D. rabiei*	Aleppo, Syria	2010	KR259382
	*Ascochyta pisi*	Pullman, USA	2007	DQ383954
	*D. pinodes*	Canberra, Australia	2009	EU338435
	*Phoma koolunga*	Canberra, Australia	2009	EU338427
	*P. medicaginis* var. *pinodella*	Palampour, India	2008	FJ032641

### Plant material

Disease responses were studied on accessions of 20 legumes species (Table 2): alfalfa (*Medicago sativa* L.), barrel medick (*M. truncatula* Gaertn.), button medick (*M. orbicularis* (L.) Bartal.), chickpea (*Cicer arietinum* L.), common bean (*Phaseolus vulgaris* L.), common vetch (*Vicia sativa* L.), faba bean (*Vicia faba* L.), fenugreek (*Trigonella foenum-graecum* L.), grass pea (*Lathyrus sativus* L.), lentil (*Lens culinaris* Medik.), oneflower vetch (*Vicia articulata* Hornem.), pea (*Pisum sativum* ssp. *sativum* L.), prinkly scorpion's tail (*Scorpiurus muricatus* L.), red clover (*Trifolium pratense* L.), soybean (*Glycine max* (L.), subterranean clover (*T. subterraneum* L.), sulla (*Hedysarum coronarium* L.), tawny pea (*P. fulvum* Sibth. & Sm.), white clover (*T. repens* L.), and white lupin (*Lupinus albus* L.). From 1 to 6 accessions per species were tested (Table 2).

**Table 2:** **Response of legume species to isolates of ***D. pinodes*** in seedling stage, measured 10 days after inoculation under controlled conditions**.

**Legume host**	**Code**	**Dp-M07-4**		**Dp-Esc-13**		**Dp-KHM-13**		**Dp-ANN-13**		**Dp-JAP-03**		**Dp-PO-03**		**Dp-CO-99**		**Dp-PdT-03**		**Dp-FR-88**		**Dl-AL10**		**Dr-Pt04**		**Df-AU04**		**Df-857**	
		**DR^A^**	**DS^B^**	**DR**	**DS**	**DR**	**DS**	**DR**	**DS**	**DR**	**DS**	**DR**	**DS**	**DR**	**DS**	**DR**	**DS**	**DR**	**DS**	**DR**	**DS**	**DR**	**DS**	**DR**	**DS**	**DR**	**DS**
**PEAS**																											
*Pisum sativum*	Messire^3^	5	80bc^*^	5	77b	5	63b	5	63ab	4.7	66bc	5	50a	5	79ab	5	50a	5	50a	0	0	0	0	2	2a	0	0
*P. sativum*	J20	5	87ab	5	93a	5	98a	5	80ab	5	100a	5	62a	5	87a	5	43a	4	35a	0	0	0	0	0	0b	0	0
*P. sativum*	J4	5	95a	5	90ab	5	75ab	5	83ab	5	100a	4.3	40a	5	57bc	5	25a	4	15b	0	0	0	0	0	0b	0	0
*P. sativum*	6NIL	5	83ab	5	93a	5	82ab	5	92a	5	77b	5	70a	5	47c	5	25a	5	30a	0	0	0	0	0	0b	0	0
*P. fulvum*	IFPI3260	4	67c	3.3	57c	4.7	60b	4	51.7b	4	42c	4.7	63a	1.3	7d	3	25a	3	22b	0	0	0	0	0	0b	0	0
AVERAGE			82A		81A		76A		74A		78A		57A		54A		30A		26A		0B		0B		0.3C		0E
**WHITE LUPIN**																											
*Lupinus albus*	Giza 2^1^	–	–	5	95a	–	–	5	40a	4	7b	5	47a	4	15a	–	–	3	13a	–	–	0	0	0	0	0	0
*L. albus*	Lup34^4^	3	20a	5	100a	5	47a	5	50a	5	40ab	4.7	43a	5	25a	3.3	40a	3	5a	–	–	0	0	0	0	0	0
*L. albus*	Lup35^4^	4	35a	5	22b	5	37ab	5	50a	5	67a	4	40a	5	20a	3	9b	3	12a	3.3	20a	0	0	0	0	0	0
*L. albus*	Lup39^4^	4.3	20a	5	25b	5	26b	5	50a	5	40ab	4.7	44a	5	25a	5	46a	3.3	15a	0	0b	0	0	0	0	0	0
AVERAGE			25CDE		53B		37B		47B		38B		43B		21.5BC		33A		9B		10B		0B		0C		0E
**CLOVERS**																											
*Trifolium pratense*	E07^5^	4.7	47a	4.3	30a	4.7	37a	5	58a	4	40a	4.7	38a	3	20a	4.7	30a	3	10a	2.7	12a	0	0	3	13a	3	8a
*T. subterraneum*	E08^5^	4	20a	3.3	20a	4	20ab	4	37a	5	32a	3.5	20a	2	17a	3	20a	3	3a	0	0b	0	0	0	0b	2.3	6a
*T. repens*	Anteria^1^	3.7	26a	4.3	30a	4.3	17b	4.7	30a	4.2	38a	3.3	15a	2	16a	5	32a	3	8a	0	0b	0	0	0	0b	2	7a
AVERAGE			31BCD		27CDE		24BC		42BC		36B	25BC			17BC		27A		7B		4B		0B		4BC		8CD
**MEDICKS**																											
*Medicago truncatula*	Parabinga^1^	–	–	3	20ab	2.7	10a	2.3	35a	5	40b	2	5b	2.7	25a	2	5a	1.3	5a	0	0	0	0	0	0	0	0
*M. truncatula*	Paraggio^1^	2	20a	2	12b	4	20a	2.3	27a	3.7	37b	2	5b	2	30a	–	–	2	6a	0	0	0	0	0	0	0	0
*M. truncatula*	M263^4^	1.7	18a	3.7	53a	2.7	10a	2	30a	4.5	80a	2.3	30a	2	37a	2	5a	1.3	11a	0	0	0	0	0	0	0	0
*M. orbicularis*	M264^4^	2	27a	–	–	2.7	7a	3	15a	4.3	37b	–	–	2.7	35a	2	10a	–	–	0	0	0	0	0	0	0	0
*M. orbicularis*	M281^4^	–	–	4.3	47a	3	10a	3.3	21a	4.5	60ab	4.7	32a	–	–	2	8a	3	15a	0	0	0	0	0	0	0	0
AVERAGE			22DEF		36BCD		12CD		25CD		38B		23C		34B		7BC		9B		0B		0B		0C		0E
**ONEFLOWER VETCH**																											
*Vicia articulata*	BGE013376^6^	4	37a	3	37a	4	27a	4.3	45ab	4	23a	2.3	23a	–	–	4.3	40a	2.7	8a	0	0	0	0	3	8a	3	4a
*V. articulata*	BGE013984^6^	4	43a	4.3	60a	3.7	30a	4	43ab	4	53a	3	25a	3	30a	3.7	28a	3	11a	0	0	0	0	0	0b	0	0a
*V. articulata*	BGE013985^6^	4	40a	3.7	63a	4.3	33a	4.3	53a	5	53a	3.7	33a	2	15a	4.3	30a	3	8a	0	0	2.3	3a	0	0b	2.3	3a
*V. articulata*	BGE018824^6^	4	43a	4	60a	4.3	32a	4	28b	4	50a	3	35a	3	20a	4.7	33a	2.7	7a	0	0	0	0	0	0b	0	0a
AVERAGE			41BC		55B		30B		43BC		48B		29BC		22BC		35A		9B		0B		0.8B		2BC		2DE
**CHICKPEA**																											
*Cicer arietinum*	ILC72^2^	3	15a	4	13a	2.7	6a	4.3	8a	1	1a	4.3	30a	0.7	2a	4.3	7a	1.3	3a	0	0b	0.3	5b	0	0	0	0
*C. arietinum*	M38^2^	3.3	10a	3.4	15a	3.7	5a	4.7	8a	3.3	10a	5	30a	4.5	25b	5	15a	1.3	2a	0	0b	4.7	40a	0	0	0	0
*C. arietinum*	AS18^2^	1.7	9a	3	8a	3	4a	–	–	3	12a	4.7	21a	3	5a	4.3	7a	1	3a	3	30a	5	37a	0	0	0	0
*C. arietinum*	AS19^2^	2.7	10a	2	13a	3	5a	5	5a	3	12a	5	28a	3	7a	5	8a	–	–	0	0b	3.8	35a	0	0	0	0
*C. arietinum*	AS23^2^	–	–	3	10a	–	–	5	7a	2.7	5a	–	–	–	–	5	10a	1	1a	0	0b	2.6	40b	0	0	0	0
AVERAGE			10EF		12EF		5D		7DE		8D	28BC			8C		10BC		2B		6B		31.4A		0C		0E
**LENTIL**																											
*Lens culinaris*	S17^2^	4	29a	4.3	62a	4	47a	4.7	53a	5	48ab	3.7	35a	–	–	3.7	15a	2	17a	4.3	24a	0	0	0	0	3	6a
*L. culinaris*	S23^2^	4	57a	3.7	60a	4.7	47a	5	67a	5	60a	2.7	25a	2.3	10a	4	33a	2	8ab	4.3	28a	0	0	0	0	1.5	3ab
*L. culinaris*	R5^2^	4	30a	4.7	57a	4	27b	4.7	37a	4	50ab	3.3	30a	3	22a	4	17a	2	4b	4	45a	0	0	0	0	0	0b
*L. culinaris*	R17^2^	4	43a	4	50a	4.5	42ab	4.7	45a	4	32b	3.7	20a	1.3	7a	3.3	7b	0.7	2b	4	48a	0	0	0	0	1.3	1b
AVERAGE			40BCD		58B		40B		49B		46B		27BC		13BC		18AB		6B		35A		0B		0C		3CDE
**SOYBEAN**																											
*Glycine max*	PI08100^5^	1.7	8	0	0	3	10	3.3	12	3	30	1	1	2	17	2	5	3	4	0	0	3	4	3.3	17	4	30
AVERAGE			8EF		0F		10CD		12DE		30BC		1D	17BC			5BC		4B		0B		4AB		17A		30A

**Table:** 

**Host specie**	**Code**	**Dp-M07-4**		**Dp-Esc-13**		**Dp-KHM-13**		**Dp-ANN-13**		**Dp-JAP-03**		**Dp-PO-03**		**Dp-CO-99**		**Dp-PdT-03**		**Dp-FR-88**		**Dl-AL10**		**Dr-Pt04**		**Df-AU04**		**Df-857**	
		**DR**^A^	**DS**^B^	**DR**	**DS**	**DR**	**DS**	**DR**	**DS**	**DR**	**DS**	**DR**	**DS**	**DR**	**DS**	**DR**	**DS**	**DR**	**DS**	**DR**	**DS**	**DR**	**DS**	**DR**	**DS**	**DR**	**DS**
**COMMON VETCH**																											
*Vicia sativa*	3151^4^	3	33a	2	15a	3.3	30a	3	13a	2	6a	3	20a	1.3	10a	2.7	10a	3	3a	0	0b	1	2a	4	5a	3.7	8a
*V. sativa*	3154^4^	3	37a	2.3	28a	3.3	35a	3	23a	2.3	18a	3	20a	2	10a	2.7	8a	3	11a	1.7	7ab	1.7	5a	4	10a	4	6a
*V. sativa*	3155^4^	3.7	33a	2.3	27a	3	35a	3	15a	2	17a	2.3	20a	1.7	10a	2.3	13a	1	3a	0.9	2b	0.5	2a	4	8a	4	8a
*V. sativa*	3156^4^	3	25a	2	23a	3.7	36a	3	23a	2	12a	2.7	13a	1.3	8a	2	8a	0.3	1b	2	10a	0	0a	4	5a	4	8a
AVERAGE			32BCD		23DEF		34B		18DE		14CD		18CD		10C		10BC		5B		4B		2B		7B		7C
**GRASS PEA**																											
*Lathyrus sativus*	ILAT1^3^	4	33a	4.7	40a	4	37a	–	–	4.7	40a	–	–	–	–	4.3	32a	2.3	4a	0	0b	0	0	0	0b	0	0
*L. sativus*	ILAT10^3^	3.7	50a	4.3	57a	3.7	17a	5	63a	4.7	37a	4.3	48a	2.3	17a	4	20a	3.5	12a	1	2a	0	0	4	5ab	0	0
*L. sativus*	ILAT16^3^	4	43a	5	53a	4	28a	5	50a	4.3	50a	3.7	33a	1.7	10a	3.7	17a	3	7a	0	0b	0	0	4	10a	0	0
*L. sativus*	ILAT18^3^	4.3	46a	4	37a	4.3	37a	5	55a	4.7	43a	4.7	48a	1	7a	4.3	23a	2.4	5a	–	–	0	0	0	0b	0	0
*L. sativus*	BGE017184^6^	4.3	42a	5	70a	2	27a	5	47a	4.7	45a	4	37a	0	0b	4	30a	3.7	11a	–	–	0	0	–	–	–	–
AVERAGE			43B		51B		29B		54B		36B		42B		13AB		26A		8B		0.6B		0B		3BC		0E
**SULLA**																											
*Hedysarum coronarium*	Sparacia^1^	2.7	20a	3.3	58a	4	43a	2.3	8	4.7	47a	3	20b	2	15a	1.7	9b	0.7	1a	0	0b	0	0	0	0	0	0
*H. coronarium*	Grimaldi^1^	1.5	10a	2.7	33a	4	45a	–	–	5	60a	3	42a	1	5b	1.7	5b	1.3	10a	0	0b	0	0	0	0	0	0

A *DR, Disease rating following 0–5 scale defined by Roger and Tivoli (1996)*.

B *DS, final disease severity (%) measured under controlled conditions*.

1 *Commercial varieties are named*.

2 *Belonging to IAS-CSIC collection*.

3 *Provided by ICARDA (Syria)*.

4 *Collected by authors*.

5 *Provided by USDA (USA)*.

6 *Provided by CRF-INIA (Spain)*.

* *Data followed with different letters, per column (lower letter types) and host species (capital letter type), are significantly different (LSD test, P = 0.01). − Not determined*.

To ensure experiments with a uniform plant development stage, seeds were scarified by nicking with a razor blade and then germinated for 48 h on wet filter paper in a Petri dish at 4°C. The Petri dishes were then transferred to 20°C for 5–7 days. Germinated seeds were planted into plastic pots (6 × 6 × 10 cm) filled with a 1:1 mixture of sand and peat in a rust-free growth chamber. Plants were pre-germinated and sown at 3 days intervals in order to be able to select seedlings at the same growing stage at the time of inoculation. There were three independent replicates per fungal isolate, arranged in a complete randomized design. Each replicate consisted of 3 pots with 5 plants each per accession. Experiments were repeated three times. Pea cv. Messire was included in each replication as a common susceptible check. Plants were grown in a growth chamber at 20°C, under a photoperiod of 14/10 h day/night regime, with 148 μmol/m^2^s irradiance at plant canopy for 3 weeks, until the plants reached the 4–5-leaf stage.

### Plant inoculation

Plants with 4-5 leaves were inoculated as described by Fondevilla et al. (2005) with some modifications. Inoculum was prepared by multiplying spores of each isolate on PDA (Potato Dextrose Agar) medium with chloramphenicol (60 mg/l PDA) and ampicillin (50 mg/l PDA) at 20°C with 16 h light/8 h dark photoperiod. Spore suspensions were prepared by flooding the surface of 10-day-old cultures with sterile distilled water, gently scraping the colony with a glass rod and filtering the suspension through two layers of sterile cheesecloth. Concentration of pycnidiospores was determined with a haemocytometer and adjusted to 10^6^ spores/ml. Tween 20 (VWR) was added as wetting agent (two drops per 500 ml pycnidiospore suspension). The pycnidiospore suspensions were sprayed at the 4–5-leaf stage using a handheld sprayer at a rate of 1 ml per plant. After inoculation, plants were covered with a polyethylene sheet during the first 24 h in darkness, and high humidity was ensured by ultrasonic humidifiers operating for 15 min every 2 h. Later on, the polyethylene cover was removed and plants were maintained 9 more days in a growth chamber (under conditions described above). Every 2 days, water was added to the trays to maintain high relative humidity (95–100%).

### Disease assessment

Plant response to infection was visually assessed 10 days after inoculation using two separate assessments. Disease severity (DS) was assessed by a visual estimation of the percent of diseased tissue per plant (Fondevilla et al., 2005). In addition, disease rating (DR) was visually assessed on leaves over the first, second and third nodes of each plant using a 0–5 scale defined by Roger and Tivoli (1996) were 0 = no lesions; 1 = a few scattered flecks; 2 = numerous flecks; 3 = 10–15% of the leaf area necrotic and appearance of coalescent necrosis; 4 = 50% of the leaf area dehydrated or necrotic; 5 = 75–100% of the leaf area dehydrated or necrotic. DR was then calculated as the average of values scored per node. Accessions displaying an average DR > 3 combined with DS > 35% were considered as highly susceptible, accessions displaying an average DR > 3 combined with DS values lower than 35% were considered as susceptible, accessions showing an average DR included between 2 and 3 combined with DS values < 35% were considered as moderately resistant and, finally, accessions displaying DR < 2 combined with DS values < 10% were considered as highly resistant.

### DNA extraction and its amplification

Monoconidial cultures of the 13 isolates were grown in Petri dishes using PDA medium as described above. Mycelium was collected by flooding the surface of 5-day-old cultures with sterile distilled water (2 ml per Petri dishes), gently scraping the colony with a glass rod and filtering the suspension through two layers of sterile cheesecloth. Three Petri dishes per isolate were used, in order to ensure sufficient amount of fungal material. Suspension was centrifuged at maximum speed (14,000 rpm) and pellet was collected. DNA was extracted from ground mycelium using the DNeasy plant minikit (Qiagen, Ltd.). DNA concentration was determined using an ND-1000 spectrophotometer (NanoDrop Technologies) and adjusted to 20 ng μl/1 for PCR. Primers ITS1 and ITS2 were used to amplify the nuclear ribosomal internal transcribed spacer (ITS) region ITS1-5.8S-ITS2 following the protocol described by White et al. (1990). PCR products were extracted with a sterile scalpel and purified using the QIAquick Gel Extraction kit (Qiagen®) following the protocol of the manufacturer. The purified products were cloned using the pGEM-T Easy Vector Systems kit (Promega, Madison, WI, USA) following Barilli et al. (2011) protocol. Sequencing was carried out on an ABI 3730 XL sequencer (Applied Biosystems, Foster City, CA, USA) at the DNA Sequencing Service, STAB VIDA GENOMICS LAB, Caparica, Portugal. For each isolate, two clones were sequenced. Both forward and reverse strands were sequenced for each clone. ITS sequences were submitted to GenBank.

In addition to this, sequences from *Ascochyta pisi, Didymella pinodes, Phoma koolunga*, and *P. medicaginis* var. *pinodella* (Table 1) retrieved from GenBank (http://www.ncbi.nih.gov; Davidson et al., 2007; Peever et al., 2007) were included in the analysis.

### Statistical analysis

#### Disease responses

All isolate x species combinations (including several accessions per species) were arranged in a completely randomized design in a controlled condition growth chamber. For the whole data set, only final disease severity values were included in the statistical analysis. Disease severity was first analyzed by taking into account differences in pathogenicity between the 13 *Didymella* spp. isolates according to the species evaluated (by averaging disease severity among accessions within each species).

Disease severity was assessed for every *Didymella* spp. isolate between accessions within each species. The whole experiment was repeated three times. Before performing analyses of variance, the normality and equality of variances were checked using Shapiro–Wilk's (Shapiro and Wilk, 1965) and Bartlett's tests (Little and Hills, 1978) respectively. When necessary, DS percentage data were transformed to angles (y = arcsine (x/100)) and again checked before applying analysis of variance. Differences between isolates, species, or accessions within species were compared by analysis of variance (ANOVA) followed by a least significant difference (LSD) test, with values of *P* < 0.01 considered significant. Statistical analyses were performed with Statistix software (version 8.0; Analytical Software, Tallahassee, USA).

Disease rating (DR) was visually estimated as the mean disease score over the first, second and third leaves of each accession within each specie.

The entire data set was analyzed by Principal Component Analysis (PCA) using the web-based software PAST (Hammer et al., 2001), available at http://nhm2.uio.no/norlex/past/download.html, with the following settings: covariance matrix type, four principal components, 1-fold change threshold for clusters, and 0.3 correlation thresholds for clusters. PCA results were represented as a biplot, with accessions more susceptible to a specific *Didymella* spp. isolate (according to both DS and DR) located in the same area of the graph.

#### ITS sequence analysis

Sequences were aligned and adjusted manually with Mega version 6 (Tamura et al., 2013) using the penalties of 15 for gap opening and 6.66 for gap extension. Estimates of genetic similarity (GS) were calculated for all possible pairs of genotypes according to Rho similarity coefficient (Posada and Crandall, 1998).

The evolutionary history was inferred using the unweighted pair-group method with arithmetic average (UPGMA; Sneath and Sokal, 1973). The evolutionary distances were computed using the Maximum Composite Likelihood method (Tamura et al., 2004) and a dendrograms was constructed.

The trees were rooted using *P. koolunga* as outgroup. The scores between 50 and 74 bootstrap percentages (BS) were defined as weak support, scores between 75 and 89% BS as moderate support and scores > 90% BS as strong support. A likelihood ratchet employs multiple sequential truncated searches on different starting trees created by fast algorithmic searches on reweighed data, in the hope of exploring a larger pro- portion of tree space, analogous to the parsimony ratchet (Nixon, 1999). We ran 200 iterations with the general time reversible likelihood model of evolution with gamma distribution (GTR+G) and uniformly reweighing 15% of the data-set per iteration. Bootstrap support values from 1000 replicates were calculated using the heuristic search with random addition-sequence with 10 replicates limited to 10,000 tree rearrangements (branch swaps) imposed separately for each addition-sequence replicate (rearlimit = 10,000; limitperrep = yes). The tree is drawn to scale, with branch lengths in the same units as those of the evolutionary distances used to infer the phylogenetic tree. The evolutionary distances are reported in the units of the number of base substitutions per site. The rate variation among sites was modeled with a gamma distribution (shape parameter = 1). All positions containing gaps and missing data were excluded in analyses.

## Results

**Figure 1:**
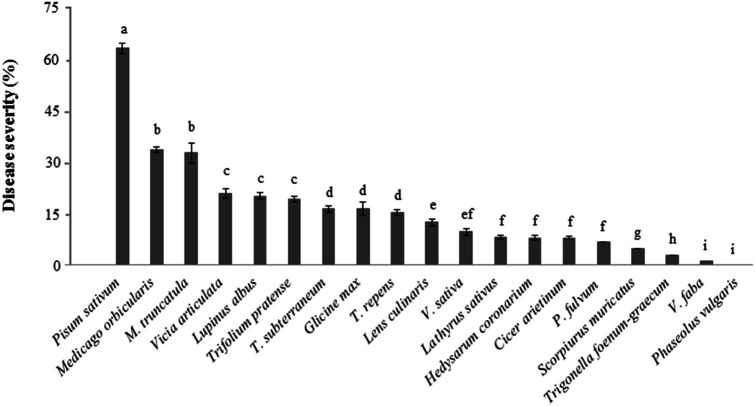
**Percentage of disease severity (DS%) measured on foliar organs of different legume species in response to inoculation with ***D. pinodes*** isolate Dp-CO-99 under controlled conditions**. Averages per species are presented. The bars indicate the standard deviation; different letters indicate significant differences (*P* = 0.01).

The local *Didymella pinodes* isolate Dp-CO-99 caused different disease rating (DR) (Table 2) as well as significantly different disease severity (DS) values on the tested legume species (*P* < 0.01; Figure 1). The highest levels of susceptibility were found in *P. sativum* (DR = 5; DS = 67%) confirming expectations (Fondevilla et al., 2005), followed by *L. albus* (DR = 4.7; DS > 20%), *Trifolium* spp., *Medicago* spp., *V. articulata, C. arietinum*, and *L. culinaris* (2 ≤ DR < 3; DS > 15%). Some infection was also observed on *G. max, V. sativa, L. sativus, H. coronarium, P. fulvum, S. muricatus, V. faba*, and *T. foenum-graecum* although at the level of resistance (DR < 2; DS < 20%). *P. vulgaris* did not showed any symptoms of fungal infection (DS and DR = 0; Figure 1).

Results from cross inoculations performed with different *Didymella* spp. showed that the legume species under study displayed differential resistance/susceptibility to each isolate as indicated by significant specie x isolate interactions in ANOVA (*P* < 0.01; Table 2). Statistical analysis showed a significant effect of legume species (sum of squares = 353,064, *P* < 0.001), fungal isolates (sum of squares = 125,118, *P* < 0.001), and their interaction (sum of squares = 75,346, *P* < 0.001), indicating that not all *D. pinodes* isolates displayed the same infection pattern toward the legume species involved in this study.

**Figure 2:**
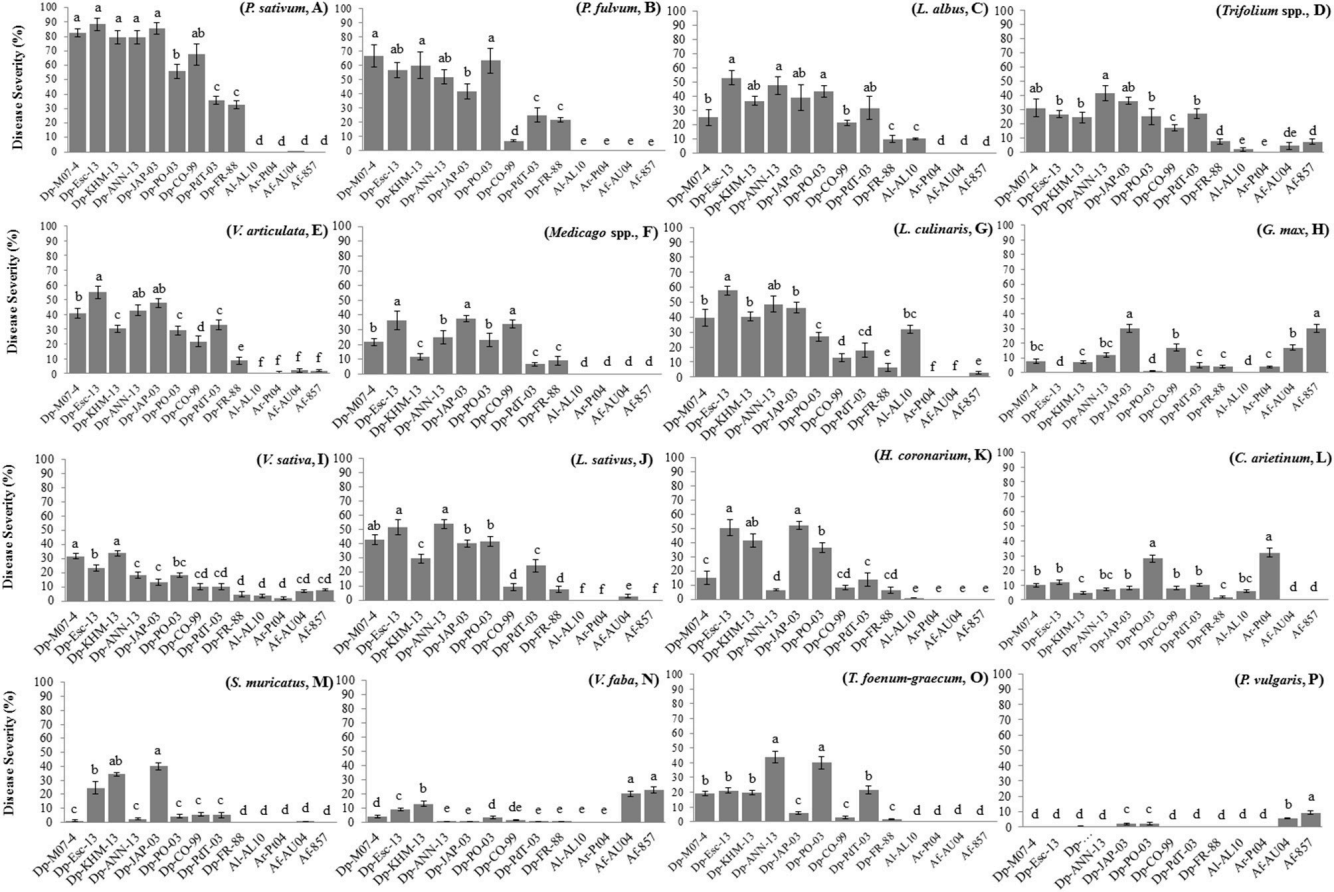
**Disease severity (%) measured on whole plants of different legume species after infection by isolates of ***Didymella*** spp. under controlled conditions**. Averages per species are presented: **(A)** pea (*Pisum sativum*), **(B)** tawny pea (*P. fulvum*), **(C)** white lupin (*Lupinus albus*), **(D)** clovers (*Trifolium pratense, T. subterraneum, T. repens*), **(E)** oneflower vetch (*V. articulata*), **(F)** medicks (*Medicago orbicularis, M. truncatula*), **(G)** lentil (*Lens culinaris*), **(H)** soybean (*Glycine max*), **(I)** common vetch (*V. sativa*), **(J)** grass pea (*Lathyrus sativus*), **(K)** sulla (*Hedysarum coronarium*), **(L)** chickpea (*Cicer arietinum*), **(M)** prinkly scorpion's tail (*Scorpiorus muricatus*), **(N)** faba bean (*Vicia faba*), **(O)** fenugreek (*Trigonella foenum-graecum*), **(P)** common bean (*Phaseolus vulgaris*). The bars indicate the standard deviation. Different letters indicate significant differences (*P* = 0.01).

*P. sativum* accessions showed DR values = 4 against all *D. pinodes* tested (Table 2), although level of infection varied greatly (DS from 15 to 100%). Isolates Dp-M07-4 (DS 80–95%), Dp-Esc-13 (DS 77–93.3%), Dp-JAP-03 (DS 66–100%), and Dp-KHM-13 (DS 63–98%) were the most aggressive on cultivated peas (Table 2, Figure 2A). *P. fulvum* was generally more resistant than *P. sativum*, with DR ranging from 1.3 to 4.7 and DS from 7 to 67%. In particular, accession IFPI3260 confirmed here its high resistance against Dp-CO-99 (DR = 1.3, DS = 6.7; Figure 2B; Fondevilla et al., 2005). In addition, *P. fulvum* was also moderately resistant to isolates Dp-FR-88 and Dp-Esc-13 (DR = 3; DS < 25%). As for *P. sativum*, accession IFPI3260 was immune to other *Didymella* spp. isolates tested (Table 2).

Accessions from *L. albus* were also susceptible to *D. pinodes* (DR = 3), showing level of infection that varied depending on the isolate tested (average DS = 34%, range 9–100%). Isolates Dp-Esc-13 and Dp-ANN-13 were the most virulent (DR = 5; DS > 40%; Table 2, Figure 2C). By contrary, *L. albus* was resistant to both *D. rabiei* and *D. fabae*, while only accession Lup35 was moderately infected by *D. lentil* (Table 2).

*Trifolium* spp. showed responses to *D. pinodes* infections that were from moderately resistant to susceptible (averages ranging between DR 2.5–4.6 and DS 7–42%; Table 2). Isolate Dp-ANN-13 was the most virulent (DR > 4.7; DS > 30%) while Dp-CO-99 and Dp-FR-88 the lesser (DR < 3.7; DS < 20%; Table 2, Figure 2D). Accessions studied were not infected by *D. rabiei*, whereas *T. pratense* was slightly infected by *D. lentil* and *D. fabae* (Table 2).

*V. articulata* accessions were from highly susceptible to moderate resistant against *D. pinodes* inoculations (averages ranging between DR 2.6–4.3 and DS 9–55%), being differences significant among accessions and isolates (*P* < 0.01) (Table 2, Figure 2E). *V. articulata* was immune to *D. lentil*, whereas only certain accessions were slightly infected by *D. rabiei* or *D. fabae* (DR from 2.3 to 3.7, DS < 8%).

Similarly, *Medicago* spp. accessions showed from resistance to susceptibility to *D. pinodes* infections. Nevertheless, differences between plant species were not consistent (Table 2, Figure 2F). Isolate Dp-JAP-03 was the most virulent on all *Medicago* accessions studied inciting DR ranging from 3.7 to 5 and DS ranging from 37 to 80%. *Medicago* accessions were not affected by any other *Didymella* spp.

Response of *L. culinaris* accessions to *D. pinodes* varied greatly, depending on the isolate tested (averages ranging between DR 0.7–5 and DS 4–67%) (Table 2, Figure 2G). As for peas, isolates Dp-Esc-13, Dp-JAP-03, and Dp-M07-4 were highly virulent on all accessions tested (DR > 3.7, DS > 40%; Table 2). By contrary, lentils were less damaged by isolate Dp-FR-88 (DR ≤ 2, DS < 10%). As expected, all accessions tested were susceptible to *D. lentil*, with no significant differences between them (DR ≥ 4, DS ≥ 20%). By contrary, *D. rabiei* did not cause any symptoms on lentils and *D. fabae* was only slightly infective (DR < 3, DS ≤ 6%; Table 2).

Accession PI08100 from *G. max* showed from moderate to high resistance against *D. pinodes* infections (Figure 2H), being isolate Dp-JAP-03 the most virulent (DR = 3, DS = 30%). By contrary, no symptoms were found on PI08100 after Dp-PO-03 and Dp-Esc-13 inoculations. This accession was immune to *D. lentil*, slightly infected by *D. rabiei* (DR = 3, DS = 4%) and susceptible to *D. fabae* (DR > 3.3, DS > 17%; Table 2).

Similarly, responses from *V. sativa* varied greatly, being resistant to isolates Dp-CO-99 and Dp-FR-88 (averages DR < 1.8 and DS < 10%) and susceptible to Dp-KHM-13 (DR > 3, DS > 30%), with no significantly difference among accessions (Table 2, Figure 2I). *V. sativa* showed a fully compatible interaction with both *D. fabae* isolates in spite of a reduced severity (DS < 10%). Nevertheless, both *D. rabiei* and *D. lentil* caused foliar symptoms at reduced rates (DR < 2, DS ≤ 10; Table 2).

Except for local isolate Dp-CO-99, studied *L. sativus* accessions were moderately or highly susceptible to all *D. pinodes* isolates studied, being isolates Dp-Esc-13 and Dp-ANN-13 the most virulent (DR > 4, DS > 37%; Table 2, Figure 2J). Accessions from *L. sativus* were immune or highly resistant to infection from *D. rabiei, D. lentil*, and *D. fabae* isolates (Table 2).

Responses of *C. arietinum* varied greatly depending both on the *D. pinodes* isolate employed as well as the accession tested (Figure 2L, Table 2), but infection was always reduced compared to pea accessions. Accessions showed DR from low to high, depending on the isolate, but always with low DS (<30 %). Isolate Dp-Po-03 was the most virulent on chickpea (DR > 4.3), while all accessions were resistant to isolate Dp-FR-88 (DR < 1.3, DS < 3%). Chickpea was resistant to both *D. fabae* isolates, while accession AS18 showed moderate susceptibility to Dl-AL10 infection. Chickpea showed a fully compatible interaction with *D. rabiei* isolate studied (Dr-Pt04) although significant differences between accessions were found (Table 2).

*H. coronarium, S. muricatus*, and *T. foenum-graecum* showed differential responses to *D. pinodes* inoculations depending principally on the isolate tested (*P* < 0.01; Figures 2K,M–O, respectively). In general, accessions showed symptoms that were significantly reduced comparing with *P. sativum*, also if some exceptions were found (e.g., *H. coronarium* and DP-JAP-03 or *T. foenum-graecum* and Dp-PO-03 with DR ≥ 4 and DS ≥ 30%; Table 2). With the exception of isolate Dp-KHM-13, *V. faba* was highly resistant against almost all *D. pinodes* studied (DR ≤ 2 and DS ≤ 10%; Table 2). Accessions belonging to *H. coronarium, S. muricatus*, and *T. foenum-graecum* were highly resistant or immune to infection with other *Didymella* spp. *V. faba* was highly susceptible to both *D. fabae* isolates with no significant differences among accessions, while no symptoms were found after Dr-Pt04 and Dl-AL10 inoculations (Table 2).

Finally, *P. vulgaris* was highly resistant to all *Didymella* spp. isolates since no or limited symptoms were foundflentils were less damaged by isolate on all accessions tested (DR ≤ 1.3, DS ≤ 2%) with exception of *D. fabae* that caused compatible interactions (DR ≥ 3.3) although with reduced DS values (Table 2, Figure 2P).

Among the isolates tested, Dp-KHM-13 was the most virulent being common bean the unique legume specie tested that was immune, while Dp-FR-88 was the lesser damaging isolate (Table 3). Isolate Dl-AL10 (*D. lentil*) was only virulent on *L. culinaris* accessions, while isolate Dr-Pt04 (*D. rabiei*) showed symptoms on *C. arietinum* and, although limited, on *G. max*. Finally, *G. max, P. vulgaris, T. pratense, V. sativa*, and *V. faba* were susceptible to isolates from *D. fabae* (Table 3).

**Table 3:** **Disease reaction of fourteen isolates of ***Didymella*** spp. from different geographical origin on fifteen leguminous species, performed under controlled growing conditions**.

**Host specie**	**N**	**Dp-M07-4**	**Dp-Esc-13**	**Dp-KHM-13**	**Dp-ANN-13**	**Dp-JAP-03**	**Dp-PO-03**	**Dp-CO-99**	**Dp-PdT-03**	**Dp-FR-88**	**Dl-AL10**	**Dr-Pt04**	**Df-AU04**	**Df-857**
*Pisum sativum*	4	++++	++++	++++	++++	++++	++++	++++	++++	++++	–	−	−	−
*P. fulvum*	1	++++	++++	++++	++++	++++	++++	–	+	+	–	–	–	–
*Lupinus albus*	4	+++/+^*^	++++/+++	++++/+++	++++	++++/+++	++++	+++	++++/+	+/+++	−/+++	–	–	–
*Trifolium pretense*	1	++++	+++	++++	++++	++++	++++	+	+++	+	+	–	+	+
*T. repens*	1	+++	+++	+++	+++	++++	+++	+	+++	+	–	–	–	+
*T. subterraneum*	1	+++	+++	+++	++++	+++	+++	+	+	+	–	–	–	+
*Lathyrus sativus*	5	++++/+++	++++	+++/++++	++++	++++	++++/+++	−/+	+++	+/+++	–	–	−/+++	–
*Lens culinaris*	4	++++/+++	++++	++++/+++	++++	++++/+++	+++/+	+/-	+++	−/+	++++/+++	–	–	−/+
*Vicia articulate*	4	++++	++++	+++	++++/+++	++++/+++	+++/+	+	+++/++++	+	–	−/+	−/+	−/+
*Trigonella foenum-graecum*	3	+++	+++/+	+++/+	++++/+++	–	+++/++++	–	−/+	–	–	–	–	–
*Medicago orbicularis*	2	+	++++	+	+++	++++	+++	+	+	+	–	–	–	–
*M. truncatula*	3	+	+/+++	+/+++	+	++++	+	+	–	−/+	–	–	–	–
*Cicer arietinum*	5	−/+++	+++/+	+/+++	+++	−/+++	+++	−/++++	+++	–	−/+	++++/−	–	–
*Hedysarum coronarium*	4	+/-	++++/+	++++	+	++++	++++/+	−/+	−/+	–	–	–	–	–
*Scorpiurus muricatus*	4	–	−/+++	−/++++	–	++++/+	–	–	–	–	–	–	–	–
*Vicia sativa*	4	+++/++++	+	+++/++++	+	+	+	–	+	−/+	–	–	+++	+++
*Glycine max*	1	–	–	+	+++	+++	–	+	–	+	–	+	+++	+++
*Vicia faba*	4	–	−/+	+	–	–	–	–	–	–	–	–	+++	+++
*Phaseolus vulgaris*	4	–	–	–	–	–	–	–	–	–	–	–	+++	+++

Results correspond to the combination of Disease Rate (based on a 0–5 scale, Roger and Tivoli 1996) and the % of the whole plant covered by symptoms (% Disease Severity, DS %) as follows:

*++++, highly susceptible (DR ≥ 3 and DS > 35%); +++, susceptible (DR ≥ 3 and DS < 35%); +, moderately resistant (2 < DR < 3 and DS < 35%); –,highly resistant (DR < 2 and DS < 10%)*.

* *When several classifications are reported means that genotypes from the same specie showed different responses to the same fungal isolate. Classification reported in first position is the most common*.

**Figure 3:**
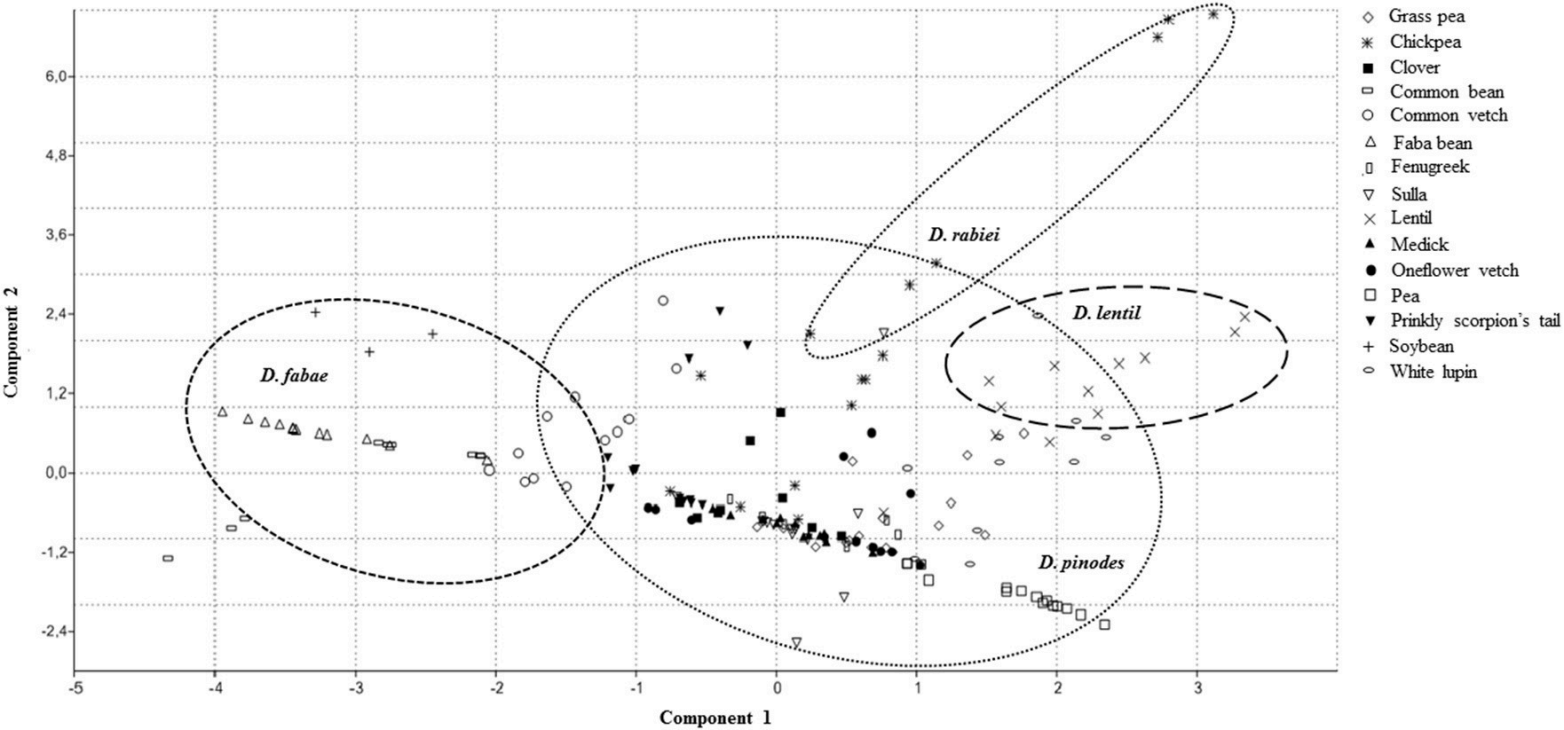
**Scattered diagram generated by principal component analysis (PCA) showing associations between Disease Severity and Disease Rating response performed by 13 isolates of ***Didymella*** spp. on 15 leguminous species**. A short distance between plant accessions and fungal isolate in the component space is indicative in susceptibility of the plant/pathogen interaction.

Principal component analysis (PCA) showed that two principal axes gave eigenvalues greater than 1, while the other axis all had eigenvalues lesser than 1 (Table 4). Hence, the first two principal components were considered important and contribute the most in the distribution of variation existing among the isolates. The component 1 had an eigenvalue of 2.8034, accounted for 40.62% of the overall variance in the data set (Table 4). Component 2 had an eigenvalue of 2.2101 and accounted for 31.1% of the total variance. Hence, the two principal components contributed for 71.69% of the total variability (Table 4). The first pc was more related to the level of aggressiveness expressed by *D. pinodes, D. lentil*, and *D. rabiei* isolates, while the second pc contributed for those expressed by *D. fabae* isolates to all cultivars tested (Figure 3). On the other hand, we can also appreciate certain host specificity between the legumes and fungal isolate species. The scattered diagram showed a major distance between isolates belonging to *D. fabae* and *D. rabiei* with the rest that were studied (Figure 3).

**Table 4:** **Principal components for disease rating (DR) and disease severity (DS) values of 13 isolates of ***Didymella*** spp**.

	**Component 1**	**Component 2**
Eigenvalues	2.8034	2.2101
Proportion of variance	40.623	31.067
Cumulative variance	40.623	71.690

**Figure 4:**
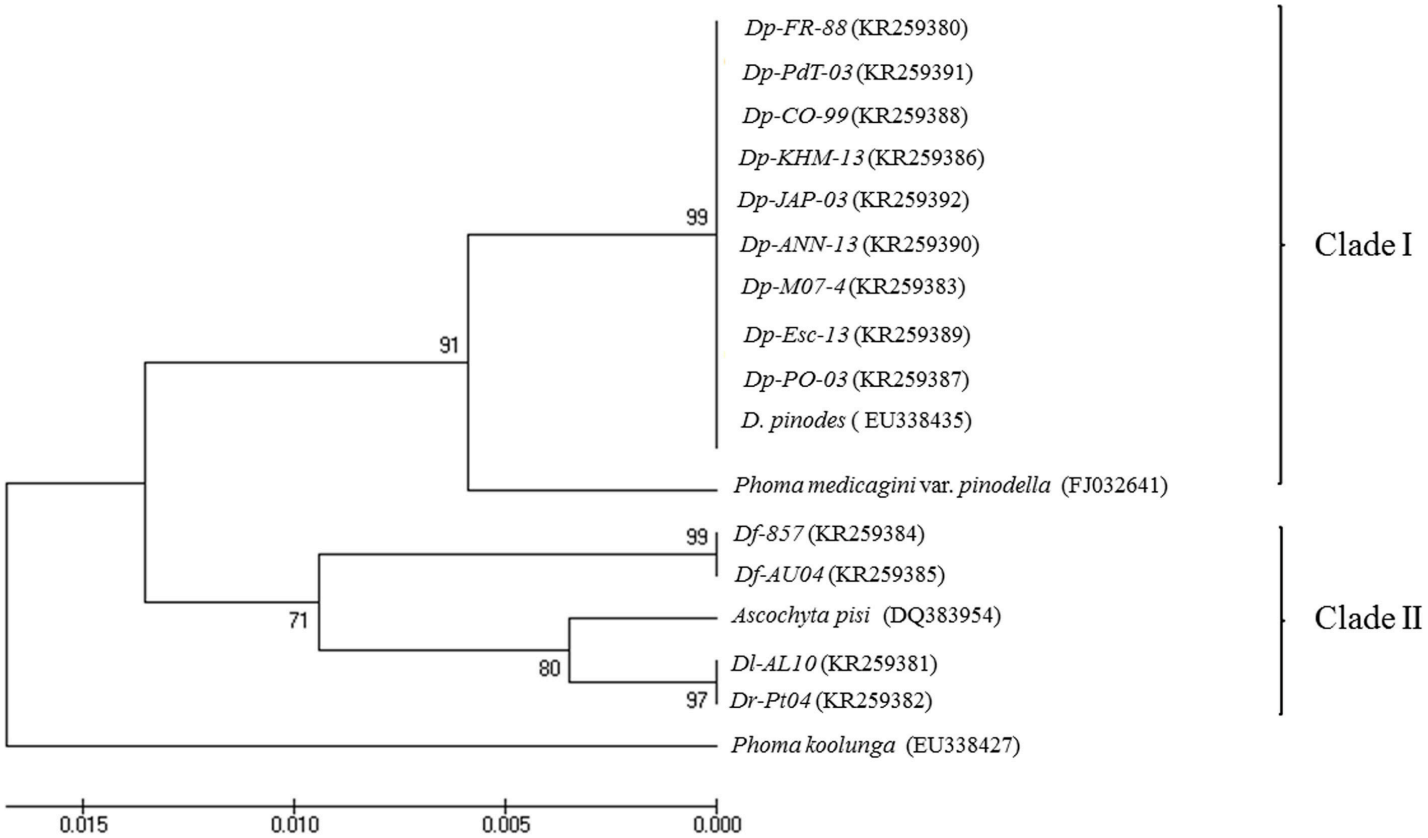
**UPGMA dendrograms of 13 samples of ***Didymella*** spp. based on Dice distance for Internal Transcribed Spacer regions analysis**.

ITS analysis by MEGA6 originates an optimal tree with the sum of branch length = 0.06595538. The percentage of replicate trees in which the associated taxa clustered together in the bootstrap test (1000 replicates) is shown next to the branches (Figure 4). The tree is drawn to scale, with branch lengths in the same units as those of the evolutionary distances used to infer the phylogenetic tree. The evolutionary distances are reported in the units of the number of base substitutions per site. The rate variation among sites was modeled with a gamma distribution (shape parameter = 1). The analysis involved 17 nucleotide sequences. All positions containing gaps and missing data were eliminated. There were a total of 437 positions in the final dataset.

From the dendrogram generated, using UPGMA with the genetic distance coefficient, the 17 isolates could be classified into two main clusters that clearly separate all isolates belonging to *D. pinodes* from the others (Figure 4). Cluster 1 (bootstrap support [BS] = 91 from Maximum Composite Likelihood analysis) included all isolates from *D. pinodes* used for the study as well as the *D. pinodes* isolate from GenBank. *D. pinodes* isolates showed to be monophyletic since they were included in a unique well-supported branch ([BS] = 99). The isolate of *P. medicaginis* var. *pinodella* was also included in this clade although it was divergent and on a branch apart from the rest of the isolates included.

Clade II ([BS] = 71) comprised two isolates of *D. fabae, one* isolate from *D. lentil*, one isolate from *D. rabiei* and *one* isolate from *A. pisi*. *D. fabae* isolates showed to be monophyletic since they were included in a unique well-supported branch ([BS] = 99). By contrary, isolates from *D. lentil* and *D. rabiei* clustered together in other strongly supported branch ([BS] = 97) where *A. pisi* was apart ([BS] = 80). Finally, isolate from *P. koolunga* did not fit with any other isolates.

## Discussion

Cool season legumes play an important role for human food and animal feed throughout the world. These crops are attacked by numerous aerial fungal pathogens that cause considerable losses in quality and quantity (Tivoli et al., 2006; Muehlbauer and Chen, 2007). The major necrotrophic fungal diseases are ascochyta blight on various grain legumes and *Didymella pinodes* was reported as the principal agent causing aschochyta blight on peas (Tivoli et al., 2006; Khan et al., 2013).

The aim of this current study was to analyse variations in the susceptibility of different legume species to *D. pinodes* compared to other *Didymella* spp., as well as to characterize the disease response of different cultivars within different legume species toward several *D. pinodes* isolates under controlled conditions. The results demonstrated that *D. pinodes* is able to cause disease in a number of legume species, that *D. pinodes* isolates from different geographical origin are differentially aggressive toward the legume species, and that cultivars within each legume species responded differentially to *D. pinodes*.

Infection of several host species is common in agrosystems leading to change in epidemic characteristics and pathogenicity. As a result, these processes will modify the survival of pathogen populations and their transmission (Woolhouse et al., 2001). In fact, variation in disease response can be significant at both the host species level as well as the host cultivar level, as was recently shown (Moussart et al., 2008; Le May et al., 2014). In the current study, cultivars from 20 different legume species were used to characterize the behavior of *D. pinodes* isolates sampled from pea. Visible symptoms caused by *D. pinodes* isolates were observed on all the legume species examined in this study, excepted with common bean. Large differences in susceptibility to *D. pinodes* were observed among the infected hosts, with *Pisum* spp. being the most susceptible, followed by *L. sativus, L. culinaris, L. albus, Medicago* spp., *Trifolium* spp., *T. foenum-graecum*, and *V. articulata*. In contrast to other *Didymella* species, *D. pinodes* appears to have the widest host range, since only accessions from lentil and chickpea were severely infected by *D. lentil* and *D. rabiei*, respectively, while *D. fabae* infected principally beans (common bean, faba bean, and soybean) and common vetch. Results for *D. lentil* and *D. rabiei* agreed with previous studies which demonstrated that artificial inoculations with Ascochyta fungi in the greenhouse and/or growth chambers are host-specific (Kaiser et al., 1997; Khan et al., 1999; Hernandez-Bello et al., 2006; Peever et al., 2007). In fact, it was previously found that *D. fabae, D. lentil*, and *D. rabiei* only diseased their respective hosts, while no visible symptoms were observed on any of the plant species other than faba bean, lentil and chickpea (Kaiser et al., 1997; Trapero-Casas and Kaiser, 2009). Nevertheless, for *D. rabiei*, Trapero-Casas and Kaiser (2009) also found that the fungus was able to survive on other leguminous or weeds, even though it did not show any visible symptoms and that this phenomenon could serve as secondary reservoirs in the absence of the natural host. In our study, isolates from *D. fabae* were highly virulent on faba bean but were also able to slightly infect other beans and vetch.

Regarding *D. pinodes* virulence, the results obtained with pea genotypes with very low levels of partial resistance were similar to those obtained by Fondevilla et al. (2005) and Le May et al. (2014) with common vetch and clover. All genotypes studied from *P. sativum* showed high susceptibility to all isolates tested, while accession IFPI3260 from *P. fulvum* (tawny pea) displayed a certain degree of partial resistance. These results confirms that only incomplete resistance is available for cultivated pea, while the highest levels of resistance are available in related *Pisum* species. In fact, sources of resistance to *D. pinodes* were recently found in accessions belonging to *P. fulvum, P. sativum* ssp. *syriacum*, and *P. sativum* ssp. *elatius* (Zhang et al., 2003; Fondevilla et al., 2005; Carrillo et al., 2013). Accession IFPI3260 showed from moderate to high resistance against 4 out of 9 *D. pinodes* isolates tested under controlled conditions. This accession was previously identified also as an important source of resistance against pea powdery mildew (*Erysiphe pisi* DC) and pea rust (*Uromyces pisi* (Pers.) Wint) (Fondevilla et al., 2007; Barilli et al., 2009) and is included in our department plant breeding programme.

*Lathyrus* has been reported as a resistant leguminous to *D. pinodes* infection firstly by Weimer (1947) who studied accessions belonging to *L. tingitanus, L. sativus* and *L. hirsutus*, followed by another relevant report (Gurung et al., 2002) which confirmed resistance of *L. sativus*, and added *L. ochrus* and *L. clymenum* as species with high degree of resistance. Nevertheless, all accessions from *L. sativus* used in our study resulted to be highly susceptible to all *D. pinodes* isolates tested under controlled conditions. Susceptibility in white lupin (*L. albus*), lentil (*L. culinaris*), fenugreek (*T. foenum-graecum*) and oneflower vetch (*V. articulata*) is described here for the first time, expanding the current knowledge of *D. pinodes*'s host range.

The almost complete absence of symptoms in common bean (*P. vulgaris*) against several *D. pinodes* isolates may indicate that this species is a non-host species or that the fungus had invaded the host tissues internally although no visible symptoms were observed. This has been previously found for *D. rabiei*, which was recovered consistently from inoculated tissue of pea without causing any visible symptoms (Trapero-Casas and Kaiser, 2009). Future histological studies will be necessary to clarify this fact.

Unlike common bean, common vetch (*V. sativa*), faba bean (*V. faba*), and soybean (*G. max*) may be defined as a host plant under conditions of high inoculum pressure, but all genotypes studied displayed a very high level of partial resistance against the set of fungal isolates tested. As the conditions used in this study were very favorable for disease development on plants, the results would require confirmation by testing under different infection conditions such as in the field since growth habit, canopy morphology, lodging and precocity can affect *D. pinodes* development (Khan et al., 2013) and plant susceptibility since it was reported that plant symptoms were more severe at plant maturity than at the seedling stage (Zhang et al., 2003). In addition, plant seasonality might also be another factor that influenced plant susceptibility in the field. Common vetch and faba bean are cool season legumes, whereas common bean and soybean are summer crops. Influences of mean temperatures and humidity on host plant susceptibility during crop development needs to be further investigated, as on *Didymella* spp. the temperatures before and after the fungal infection period affected disease development and symptom expression (Trapero-Casas and Kaiser, 1992; Roger et al., 1999; Frenkel et al., 2008).

The use of faba bean has been previously tested in pea intercropped field as an alternative control measure to limit aschochyta blight (Fernández-Aparicio et al., 2010), leading to a fungal reduction by up to 60%. Introduction of species as common bean, common vetch, faba bean, soybean in pea rotation or intercropped may be tested in relation with a reduction of aerial spores during the cropping season and the survival of the pathogen into the soil residues by chlamydospore and sclerotium production. In fact it has been previously reported that introduction of plants with modified characteristics than pea imposes a non-host barrier, and as a consequence, less conidia are surviving and successfully transported to new developing host tissue (Zhang et al., 2003; McDonald and Peck, 2009; Fernández-Aparicio et al., 2010).

The existence of susceptible, partially and highly resistant genotypes within the same species (as in medicks, sulla, fenugreek, chickpea, prinkly scorpion's tail) suggest that the reaction may therefore be described as cultivar specific since the fungal ability to infect these other species depends on the susceptibility of the cultivar chosen (Moussart et al., 2008). *C. arietinum* accessions showed different degrees of susceptibility depending on the accession and the isolate tested, nevertheless cv. ILC72 was one of the lesser diseased after *D. pinodes* inoculation. ILC72 is a *D. rabiei* resistant line from ICARDA which showed a degree of resistance in the field and in controlled environments (Muehlbauer and Chen, 2007), as confirmed here. This accession has been thoroughly used in breeding programmes worldwide, as well in studies of the genetic of resistance to aschochyta blight (Cobos et al., 2006; Muehlbauer and Chen, 2007; Madrid et al., 2014). Susceptibility found here to certain *D. pinodes* isolates in cultivars belonging to *H. coronarium, Medicago* spp., *S. muricatus* and *T. foenum-graecum* is also described here for the first time. The susceptibility of these pasture legume species need to be tracked under field conditions before to become a serious agricultural problem. Thus, for each legume species, it should be interesting to enlarge the set of genotypes tested to make possible the identification of resistant genotypes.

In terms of pathogenicity, results on peas showed that the local isolate Dp-Co-99 was not always the most aggressive. In fact, disease severity measured on the primary host plants showed that isolates Dp-M07-4, Dp-Esc-13, Dp-KHM-13, and Dp-ANN-13 (from Perth, Australia, Escacena del Campo, Spain and both Khemis Miliana and Annaba from Algeria, respectively) were significantly more aggressive, hence dangerous if introduced in other fields. Migration of invasive organisms might lead to selective emergence of adapted isolates in novel geographic regions and on specific host genotypes (Leo et al., 2015). The evolutionary potential of pathogens may be increased and subsequently adapt to overcome host resistances (Linde et al., 2009). Available resistance to *D. pinodes* is partial and governed by multiple quantitative resistance loci (Rubiales and Fondevilla, 2012). Pathogen aggressiveness could incur a gradual evolution and adaptation that may lead to an “erosion” of resistance, especially if a monoculture farming system is applied (Gandon, 2002).

*D. pinodes* is a teleomorph of *A. pinodes* that reproduces asexually by pycnidia containing splash-dispersed pycnospores (Roger and Tivoli, 1996), and sexually by perithecia releasing wind-dispersed ascospores (Tivoli and Banniza, 2007). With the presence of sexual reproduction, new combination of genes could arise in the field, from one growing season to the next (Ali et al., 1994). The existence of pathotypes between *D. pinodes* isolates is still a matter of concern since there are numerous reports analyzing differential reaction of fungal isolate collection on various hosts leading to ambiguous conclusions (Ali et al., 1978; Zhang et al., 2003; Setti et al., 2009, 2011). Here, despite their large geographical distance (Africa, Australia and Europe), we found a similarity between the host range pattern and the low genetic variability between the *D. pinodes* isolates used for the study. Both results from *D. pinodes* host range as well as molecular ITS analysis indicate a lack of pathotypes within the fungal collection used here.

## Conclusions

Knowledge of the host range is important to determine whether other crops could be affected. Understanding of population diversity and identification of pathogenic variation within plant species will assist in the management of ascochyta blight diseases.

If common bean is a non-host to *D. pinodes* as our results suggest, the use of this specie may have positive effect on soil infestation and subsequent disease development. Conversely, the use of grass pea, clover, lentil, oneflower vetch, white lupin might considerably increase the inoculum potential of the soil, having a deleterious effect on the subsequent pea crop. Ascospores produced in pseudothecia on overwintered debris of alternative hosts may serve as important sources of primary inoculum and/or inoculum necessary for secondary infections later in the growing season, as other aschochyta species did (Trapero-Casas et al., 1996; Trapero-Casas and Kaiser, 2009). Infected alternative hosts also may aid in the pathogen's survival from one growing season to the next, as do pea debris and infected seeds (Kaiser, 1990, 1992, 1997).

The use of chickpea, medick, sulla or fenugreek cultivars with qualitative resistance could be considered, but studies on the risk of resistance breakdown are required. As well, it would be important to determine if and which species could act as bridging hosts allowing for the crossing of *D. pinodes* isolates from one legume with those from another, as demonstrated with *Ascochyta* spp. by Hernandez-Bello et al. (2006) for *A. pisi* and *A. fabae* isolates. This is especially important in light of the plasticity of *D. pinodes* which is highly adaptable under the influence of biotic and abiotic factors (Le May et al., 2014).

## Author contributions

EB designed experiments. Together with MC carried out the experimental work. EB carried out most of the data analysis and contributed to the writing of the manuscript. DR contributed to the interpretation of results and writing of the manuscript. MC also contributed to critical reading.

### Conflict of interest statement

The authors declare that the research was conducted in the absence of any commercial or financial relationships that could be construed as a potential conflict of interest.

## References

[B1] Ali S. M. Nitschke L. F. Dube A. J. Krause M. R. Cameron B. (1978). Selection of pea lines for resistance to pathotypes of *Ascochyta pinodes, Ascochyta pisi* and *Phoma medicaginis* var. *pinodella*. Aust. J. Agr. Res. 29, 841–849. 10.1071/AR9780841

[B2] Ali S. M. Sharma B. Ambrose M. J. (1994). Current status and future strategy in breeding pea to improve resistance to biotic and abiotic stresses. Euphytica 73, 115–126. 10.1007/BF00027188

[B3] Bailey K. L. Gossen B. D. Lafond G. R. Watson P. R. Derksen D. A. (2001). Effect of tillage and crop rotation on root and foliar diseases of wheat and pea in Saskatchewan from 1991 to 1998: univariate and multivariate analyses. Can. J. Plant Sci. 81, 789–803. 10.4141/P00-152

[B4] Barilli E. Sillero J. C. Moral A. Rubiales D. (2009). Characterization of resistance response of pea (*Pisum* spp.) against rust (*Uromyces pisi*). Plant Breed. 128, 665–670. 10.1111/j.1439-0523.2008.01622.x

[B5] Barilli E. Satovic Z. Sillero J. C. Rubiales D. Torres A. M. (2011). Phylogenetic analysis of *Uromyces* species infecting grain and forage legumes by sequence analysis of nuclear ribosomal Internal Transcribed Spacer region. J. Phytopathol. 159, 137–145. 10.1111/j.1439-0434.2010.01736.x

[B6] Bretag T. W. (2004). Review: Ascochyta Blight in Field Peas. Horsham, VIC: Victorian Department of Primary Industries.

[B7] Carrillo E. Rubiales D. Pérez-de-Luque A. Fondevilla S. (2013). Characterization of mechanisms of resistance against *Didymella pinodes* in *Pisum* spp. Eur. J. Plant Pathol. 135, 761–769. 10.1007/s10658-012-0116-0

[B8] Cobos M. J. Rubio J. Strange R. N. Moreno M. T. Gil J. Millan T. (2006). A new QTL for *Ascochyta blight* resistance in a RIL population derived from an interspecific cross in chickpea. Euphytica 149, 105–111. 10.1007/s10681-005-9058-3

[B9] Davidson J. A. Hartley D. Priest M. Krysinska-Kaczmarek M. Herdina H. McKay A.. (2007). A new species of *Phoma* causes *Ascochyta blight* symtoms on field peas (*Pisum sativum*) in South Australia. Mycologia 101, 120–128. 10.3852/07-199 19271674

[B10] FAOSTAT (2015). FAOSTAT, Production, Crops. Food and Agriculture Organization of the United Nations. Available online at: http://faostat.fao.org/site/567/DesktopDefault.aspx?PageID=567#ancor (Accessed 30.09.15).

[B11] Fernández-Aparicio M. Amri M. Kharrat M. Rubiales D. (2010). Intercropping reduces *Mycosphaerella pinodes* severity and delays upward progress on the pea plant. Crop Prot. 29, 744–750. 10.1016/j.cropro.2010.02.013

[B12] Fondevilla S. Avila C. M. Cubero J. L. Rubiales D. (2005). Response to *Mycosphaerella pinodes* in a germplasm collection of *Pisum* spp. Plant Breeding 124, 313–315. 10.1111/j.1439-0523.2005.01104.x

[B13] Fondevilla S. Cubero J. I. Rubiales D. (2007). Inheritance of resistance to *Mycosphaerella pinodes* in two wild accessions of *Pisum*. Eur. J. Plant Pathol. 119, 53–58. 10.1007/s10658-007-9146-4

[B14] Frenkel O. Sherman A. Abbo S. Shtienberg D. (2008). Different ecological affinities and aggressiveness patterns among *Didymella rabiei* isolates from sympatric domesticated chickpea and wild *Cicer judaicum*. Phytopathology 98, 600–608. 10.1094/PHYTO-98-5-0600 18943229

[B15] Gandon S. (2002). Local adaptation and the geometry of host-parasite coevolution. Ecol. Lett. 5, 246–256. 10.1046/j.1461-0248.2002.00305.x

[B16] Gossen B. D. Derksen D. A. (2003). Impact of tillage and crop rotation on *Aschochyta blight* (*Ascochyta lentis*) of lentil. Can. J. Plant Sci. 83, 411–415. 10.4141/P02-088

[B17] Gurung A. M. Pang E. C. K. Taylor P. W. J. (2002). Examination of *Pisum* and *Lathyrus* species as sources of *Ascochyta blight* resistance for field pea (*Pisum sativum*). Australas. Plant Path. 31, 41–45. 10.1071/AP01069

[B18] Hammer Ø. Harper D. A. T. Ryan P. D. (2001). PAST: Paleontological Statistics Software Package for Education and Data Analysis. Palaeontologia Electronica 4. 9. Avaliable online at: http://palaeo-electronica.org/2001_1/past/issue1_01.htm

[B19] Hernandez-Bello M. A. Chilvers M. I. Akamatsu H. Peever T. L. (2006). Host specificity of *Ascochyta* spp. infecting legumes of the *Viciae* and *Cicerae* tribes and pathogenicity of an interspecific hybrid. Phytopathology 96, 1148–1156. 10.1094/PHYTO-96-1148 18943504

[B20] Jensen E. S. Peoples M. B. Boddey R. M. Gresshoff P. M. Henrik H. N. Alves B. J. R. (2012). Legumes for mitigation of climate change and the provision of feedstock for biofuels and biorefineries. A review. Agron. Sustain. Dev. 32, 329–364. 10.1007/s13593-011-0056-7

[B21] Kaiser W. J. (1990). Host range of the *Ascochyta blight* pathogen of chickpea. Phytopathology 80, 889–890.

[B22] Kaiser W. J. (1992). Fungi associated with the seeds of commercial lentils from the U.S. Pacific Northwest. Plant Dis. 76, 605–610. 10.1094/PD-76-0605

[B23] Kaiser W. J. (1997). Inter- and intranational spread of *aschochyta* pathogens of chickpea, faba bean, and lentil. Can. J. Plant Pathol. 19, 215–224. 10.1080/07060669709500556

[B24] Kaiser W. J. Wang B. C. Rogers J. D. (1997). *Ascochyta fabae* and *A. lentis*: Host specificity, teleomorphs (*Didymella*), hybrid analysis, and taxonomic status. Plant Dis. 81, 809–816. 10.1094/PDIS.1997.81.7.809 30861899

[B25] Khan M. S. A. Ramsey M. D. Scott E. S. (1999). Host range studies with an Australian isolate of *Ascochyta rabiei*. Aus. J. Agric. Res. 28, 170–173. 10.1071/ap99028

[B26] Khan T. N. Timmerman-Vaughan G. M. Rubiales D. Warkentin T. D. Siddique K. H. M. Erskine W. (2013). *Didymella pinodes* and its management in field pea: challenges and opportunities. Field Crops Res. 148, 61–77. 10.1016/j.fcr.2013.04.003

[B27] Le May C. Guibert M. Baranger A. Tivoli B. (2014). A wide range of cultivated legume species act as alternative hosts for the pea *aschochyta blight* fungus, *Didymella pinodes*. Plant Pathol. 63, 877–887. 10.1111/ppa.12154

[B28] Leo A. E. Ford R. Linde C. C. (2015). Genetic homogeneity of a recently introduced pathogen of chickpea, *Ascochyta rabiei*, to Australia. Biol. Invasions 17, 609–623. 10.1007/s10530-014-0752-8

[B29] Linde C. C. Zala M. McDonald B. A. (2009). Molecular evidence for recent founder populations and human-mediated migration in the barley scald pathogen *Rhynchosporium secalis*. Mol. Phylogenet. Evol. 51, 454–464. 10.1016/j.ympev.2009.03.002 19289174

[B30] Little T. M. Hills F. J. (1978). Agricultural Experimentation: Design and Analysis. 350 New York, NY: Wiley.

[B31] Madrid E. Barilli E. Gil J. Huguet T. Gentzbittel L. Rubiales D. (2014). Detection of partial resistance quantitative trait loci against *Didymella pinodes* in *Medicago truncatula*. Mol. Breed. 33, 589–599. 10.1007/s11032-013-9976-z

[B32] McDonald G. K. Peck D. (2009). Effects of crop rotation, residue retention and sowing time on the incidence and survival of *Ascochyta blight* and its effect on grain yield of field peas (*Pisum sativum* L.). Field Crop Res. 111, 11–21. 10.1016/j.fcr.2008.10.001

[B33] Moussart A. Even M. N. Tivoli B. (2008). Reaction of genotypes from several species of grain and forage legumes to infection with a French pea isolate of the oomycete *Aphanomyces euteiches*. Eur. J. Plant Pathol. 122, 321–333. 10.1007/s10658-008-9297-y

[B34] Muehlbauer F. J. Chen W. (2007). Resistance to *Aschochyta blights* of cool season food legumes. Eur. J. Plant Pathol. 119, 135–141. 10.1007/s10658-007-9180-2

[B35] Nixon K. C. (1999). The Parsimony Ratchet, a new method for rapid parsimony analysis. Cladistics 15, 407–414. 10.1111/j.1096-0031.1999.tb00277.x 34902938

[B36] Omri Benyoussef N. Le May C. Mlayeh O. Kharrat M. (2012). First report of *Didymella fabae*, teleomorph of *Ascochyta fabae*, on faba bean crop debris in Tunisia. Phytopathol. Mediterr. 51, 369–373.

[B37] Pande S. Siddique K. H. M. Kishore G. K. Bayaa B. Gaur P. M. Gowda C. L. L. (2005). Ascochyta blight of chickpea (*Cicer arietinum* L.): a review of biology, pathogenicity, and disease management. Aust. J. Agr. Res. 56, 317–332. 10.1071/AR04143

[B38] Peever T. L. Barve M. P. Stone L. J. (2007). Evolutionary relationships among *Ascochyta* species infecting wild and cultivated hosts in the legume tribes *Cicereae* and *Vicieae*. Mycologia 99, 59–77. 10.3852/mycologia.99.1.59 17663124

[B39] Posada D. Crandall K. A. (1998). Modeltest: testing the model of DNA substitution. Bioinformatics 14, 817–818. 10.1093/bioinformatics/14.9.817 9918953

[B40] Roger C. Tivoli B. (1996). Spatio-temporal development of pycnidia and perithecia and dissemination of spores of *Mycosphaerella pinodes* on pea (*Pisum sativum*). Plant Pathol. 45, 518–528. 10.1046/j.1365-3059.1996.d01-139.x

[B41] Roger C. Tivoli B. Huber L. (1999). Effects of interrupted wet periods and different temperatures on the development of *Ascochyta blight* caused by *Mycosphaerella pinodes* on pea (*Pisum sativum*) seedlings. Plant Pathol. 48, 10–18. 10.1046/j.1365-3059.1999.00311.x

[B42] Rubiales D. Fondevilla S. (2012). Future prospects for *Aschochyta blight* resistance breeding in cool season food legumes. Front. Plant Sci. 3:27. 10.3389/fpls.2012.00027 22645577 PMC3355812

[B43] Salam M. U. Galloway J. MacLeod W. J. Davidson J. A. Seymour M. Pritchard I. (2011). Blackspot manager model predicts the maturity and release of ascospores in relation to *Ascochyta blight* on field pea. Australas. Plant Path. 40, 621–631. 10.1007/s13313-011-0035-0

[B44] Sattar A. (1934). A comparative study of the fungi associated with blight diseases of certain cultivated leguminous plants. T. Br. Mycol. Soc. 18, 276–301. 10.1016/S0007-1536(34)80013-7

[B45] Shapiro S. S. Wilk M. B. (1965). An analysis of variance test for normality (complete samples). Biometrika 52, 591–611. 10.1093/biomet/52.3-4.591

[B46] Setti B. Bencheikh M. Henni J. Neema C. (2009). Comparative aggressiveness of *Mycosphaerella pinodes* on peas from different regions in western Algeria. Phytopathol. Mediterr. 48, 195–204. 10.14601/Phytopathol_Mediterr-2787

[B47] Setti B. Bencheikh M. Henni J. Neema C. (2011). Morphological and virulence variation among isolates of *Mycosphaerella pinodes* the causal agent of pea leaf blight. Afr. J. Agric. Res. 6, 1067–1075.

[B48] Siddique K. M. Johansen C. Turner N. Jeuffroy M.-H. Hashem A. Sakar D. (2012). Innovations in agronomy for food legumes. A review. Agron. Sust. Devel. 32, 45–64. 10.1007/s13593-011-0021-5

[B49] Sneath P. H. A. Sokal R. R. (1973). Numerical taxonomy: the principles and practice of numerical classification. Syst. Zool. 24, 263–268.

[B50] Sprague R. (1929). Host range and life history studies of some leguminous ascochytae. Phytopathology 19, 917–932.

[B51] Tamura K. Nei M. Kumar S. (2004). Prospects for inferring very large phylogenies by using the neighbour-joining method. Proc. Natl. Acad. Sci. U.S.A. 101, 11030–11035. 10.1073/pnas.0404206101 15258291 PMC491989

[B52] Tamura K. Stecher G. Peterson D. Filipski A. Kumar S. (2013). MEGA6: molecular evolutionary genetics analysis version 6.0. Mol. Biol. Evol. 30, 2725–2729. 10.1093/molbev/mst197 24132122 PMC3840312

[B53] Taylor P. W. J. Ford R. (2007). Diagnostics, genetic diversity and pathogenic variation of aschochyta blight of cool season food and feed legumes. Eur. J. Plant Pathol. 119, 127–133. 10.1007/s10658-007-9177-x

[B54] Tivoli B. Baranger A. Avila C. M. Banniza S. Barbetti M. Chen W. (2006). Screening techniques and sources of resistance to foliar diseases caused by major necrotrophic fungi in grain legumes. Euphytica 147, 223–253. 10.1007/s10681-006-3131-4

[B55] Tivoli B. Banniza S. (2007). Comparison of the epidemiology of *Ascochyta blights* on grain legumes. Eur. J. Plant Pathol. 119, 59–76. 10.1007/s10658-007-9117-9

[B56] Tran H. S. You M. P. Khan T. N. Barbetti M. J. (2016). Pea black spot disease complex on field pea: dissecting the roles of the different pathogens in causing epicotyl and root disease. Eur. J. Plant Pathol. 144, 595–605. 10.1007/s10658-015-0798-1

[B57] Trapero-Casas A. Kaiser W. J. (1992). Influence of temperature, wetness period, plant age, and inoculum concentration on infection and development of *Ascochyta blight* of chickpea. Phytopathology 82, 589–596. 10.1094/Phyto-82-589

[B58] Trapero-Casas A. Kaiser W. J. (2009). Alternative hosts and plant tissues for the survival, sporulation and spread of the *Ascochyta blight* pathogen of chickpea. Eur. J. Plant Pathol. 125, 573–587. 10.1007/s10658-009-9507-2

[B59] Trapero-Casas A. Navas-Cortés J. A. Jiménez-Díaz R. M. (1996). Airborne ascospores of *Didymella rabiei* as a major primary inoculum for *Ascochyta blight* epidemics in chickpea crops in southern Spain. Eur. J. Plant Pathol. 102, 237–245. 10.1007/BF01877962

[B60] Weimer J. L. (1947). Resistance of *Lathyrus* spp. and *Pisum* spp. to *Ascochyta pinodella* and *Mycosphaerella pinodes*. J. Agric. Res. 75, 181–190.

[B61] White T. J. Bruns T. Lee S. Taylor J. (1990). Amplification and direct sequencing of fungal ribosomal RNA genes for phylogenetics, in PCR Protocols: a Guide to Methods and Applications, eds Innis M. A. Gelfand D. H. Sninsky J. J. White T. J. (San Diego, CA: Academic Press), 315–322.

[B62] Woolhouse M. E. J. Taylor L. H. Haydon D. T. (2001). Population biology of multihost pathogens. Science 292, 1109–1112. 10.1126/science.1059026 11352066

[B63] Zhang J. X. Fernando W. G. D. Xue A. G. (2003). Virulence and genetic variability among isolates of *Mycosphaerella pinodes*. Plant Dis. 87, 1376–1383. 10.1094/PDIS.2003.87.11.1376 30812557

